# Rtt107/Esc4 binds silent chromatin and DNA repair proteins using different BRCT motifs

**DOI:** 10.1186/1471-2199-7-40

**Published:** 2006-11-09

**Authors:** David C Zappulla, Arindel SR Maharaj, Jessica J Connelly, Rebecca A Jockusch, Rolf Sternglanz

**Affiliations:** 1 Department of Biochemistry and Cellular Biology, Stony Brook University, Stony Brook, NY 11794 USA

## Abstract

**Background:**

By screening a plasmid library for proteins that could cause silencing when targeted to the *HMR *locus in *Saccharomyces cerevisiae*, we previously reported the identification of Rtt107/Esc4 based on its ability to establish silent chromatin. In this study we aimed to determine the mechanism of Rtt107/Esc4 targeted silencing and also learn more about its biological functions.

**Results:**

Targeted silencing by Rtt107/Esc4 was dependent on the *SIR *genes, which encode obligatory structural and enzymatic components of yeast silent chromatin. Based on its sequence, Rtt107/Esc4 was predicted to contain six BRCT motifs. This motif, originally identified in the human breast tumor suppressor gene *BRCA1*, is a protein interaction domain. The targeted silencing activity of Rtt107/Esc4 resided within the C-terminal two BRCT motifs, and this region of the protein bound to Sir3 in two-hybrid tests. Deletion of *RTT107/ESC4 *caused sensitivity to the DNA damaging agent MMS as well as to hydroxyurea. A two-hybrid screen showed that the N-terminal BRCT motifs of Rtt107/Esc4 bound to Slx4, a protein previously shown to be involved in DNA repair and required for viability in a strain lacking the DNA helicase Sgs1. Like *SLX *genes, *RTT107ESC4 *interacted genetically with *SGS1*; *esc4*Δ *sgs1*Δ mutants were viable, but exhibited a slow-growth phenotype and also a synergistic DNA repair defect.

**Conclusion:**

Rtt107/Esc4 binds to the silencing protein Sir3 and the DNA repair protein Slx4 via different BRCT motifs, thus providing a bridge linking silent chromatin to DNA repair enzymes.

## Background

Transcriptional silencing in the budding yeast *Saccharomyces cerevisiae *occurs at the silent mating-type loci *HMR *and *HML*, telomeres, and at the rDNA locus. At all of these silenced regions, DNA binding proteins recognize specific motifs and recruit a silencing protein complex (reviewed in [1]). *HMR *and *HML *are flanked by *E *and *I *"silencers." Each silencer has binding sites for ORC, and Rap1 or Abf1. The potent *HMR-E *silencer has a binding site for all three proteins. At telomeres, Rap1 also contributes a critical DNA-binding function, binding to the TG_1–3 _repeats.

At both the silent mating loci and at telomeres, the DNA-binding proteins recruit a Sir protein complex that can spread to silence genes at a distance (reviewed in [2]). At *HMR-E*, for example, this is achieved by ORC recruitment of Sir1 via a Sir1-Orc1 interaction [3], and Rap1 and Abf1 binding to Sir4 and Sir3 [4,5]. Sir4 and Sir3 multimerize, both with themselves and each other [4,6]. Sir4 also binds Sir2, and Sir2 plays a crucial role in the spreading of a Sir2, Sir3, Sir4 complex on chromatin by deacetylating histone H4 lysine 16. The deacetylation produces a novel product, 2' *O*-acetyl-ADP-ribose [7-9]. This compound has recently been shown to produce a conformational change in Sir3 that is likely to promote spreading of the Sir complex. Sir2, Sir3 and Sir4 are essential for silencing at the *HM *loci and at telomeres, while Sir1 plays a prominent role in silencing at the mating type loci but not at telomeres. The Yku70/Yku80 heterodimer that binds to DNA ends plays an important role in silencing at telomeres, while being dispensable for silencing at *HM *loci [10].

**Figure 1:**
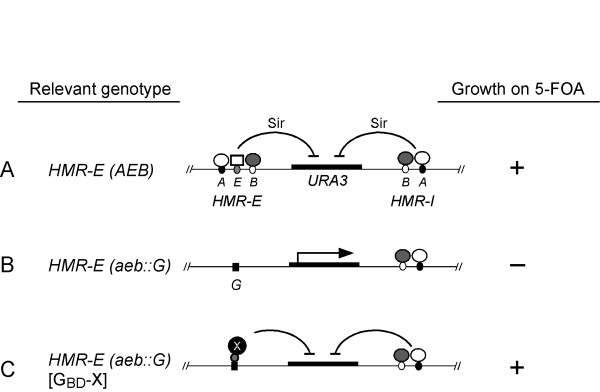
**Esc4 was identified in an *HMR *targeted silencing screen**. (A) In the presence of both wild-type silencers, *HMR-E *and *HMR-I*, the *URA3 *reporter gene at *HMR *is completely silenced in a *SIR*-dependent manner leading to growth in the presence of the drug 5-FOA. (B) When the *HMR-E *silencer is deleted (and here replaced with a binding site for the Gal4 protein (G)), the *HMR *locus is no longer silenced and the *URA3 *reporter gene is expressed. This loss of silencing at *hmr::URA3 *leads to no growth on 5-FOA media. (C) When the strain in (B) is transformed with a G_BD _plasmid (G_BD_-X) capable of causing targeted silencing, this leads to restoration of silencing at *hmr::URA3 *and growth on 5-FOA. In this way, a G_BD _hybrid protein library was screened for factors capable of targeted silencing. A G_BD_-Esc4(1–1070) hybrid was identified.

Deletion of the *HMR-E *silencer leads to loss of silencing of genes at the *HMR *locus [11,12]. However, if heterologous DNA binding sites (such as Gal4 sites) are integrated in place of *HMR-E *and the strain is transformed with certain Gal4 DNA binding domain (G_BD_) – silencing protein hybrids, silencing can be restored due to targeting of Sir proteins or proteins that bind to Sir proteins to *HMR-E *([13]; see also Figure 1). This so-called "targeted silencing" has been a useful tool for investigating the process of Sir protein recruitment to silenced loci [3,13-17]. We previously described a screen for proteins capable of targeted silencing at *HMR *[16]. In this screen many known proteins were identified, as well as several proteins not characterized at the time, which we called Esc proteins because they establish silent chromatin. One of these was Esc4 whose characterization we describe in this report. *RTT107/ESC4 *was also identified in screens for mutants with increased Ty transposon mobility or DNA repair defects [18-20]. For simplicity we will only use the name *ESC4 *in the remainder of this paper. Esc4 has been shown to be phosphorylated by Mec1 kinase on SQ/TQ motifs in response to DNA damage during S phase [21]. The phosphorylation by Mec1 has quite recently been proposed to be regulated by Slx4, which was also shown to form a complex containing Esc4 [22].

In this report we show that Esc4 has 6 BRCT motifs and that they are important for its function. Many proteins that function in repair, and even a silencing protein, Rap1, contain BRCT (BRCA1 C-terminus) motifs. This motif was first identified by database searching using the C-terminus of the human breast cancer susceptibility protein, BRCA1 [23]. Since then, BRCT motifs from the human DNA repair protein XRCC1 and the two tandem BRCT motifs of BRCA1 protein have been crystallized [24,25] and crystal and/or solution structures have also been solved of BRCTs from 53BP1, DNA ligase III, and an NAD-dependent DNA ligase [26-29]. More recently, it was discovered that BRCT motifs could specifically bind to phosphoserine-containing proteins [30,31] and structures of such complexes were subsequently also determined [32-34]. Overall, BRCT motifs are thought to mediate a diverse array of protein-protein interactions, binding to proteins with different structures, as well as to other BRCTs, both inter- and intra-molecularly (reviewed in [35]).

DNA repair requires many DNA-modifying enzymes such as nucleases, ligases, topoisomerases, polymerases and helicases. *Saccharomyces cerevisiae SGS1 *is a member of the RecQ family of genes encoding DNA helicases. In addition to *Escherichia coli recQ*, this helicase gene family includes human *BLM*, *WRN*, *RECQL*, and *Schizosaccharomyces pombe rqh1*^+^. Members of the RecQ family of helicases have been implicated in genomic stability, aging and cancer. Yeast *SGS1 *has been shown by several labs to suppress DNA recombination, gross chromosomal rearrangements, and Ty1 transposition, and to exhibit 3' to 5' helicase activity [20,36-38]. Six *SLX *genes have been isolated in a synthetic lethal screen using an *sgs1*Δ mutant [39]. Two of these genes, *SLX2 *and *SLX3*, encode the Mus81/Mms4 nuclease that acts on branched DNA structures. *SLX1 *is also predicted to be a nuclease, based on its sequence [40]. Slx1 and Slx4 coimmunoprecipitate and *slx1 *and *slx4 *mutants display similar phenotypes and have been proposed to function together [39]. In addition, a genome-wide genetic interaction (synthetic genetic array, SGA) screen isolated a total of 24 genes that show a synthetic growth interaction with an *sgs1*Δ mutation [41]. This screen identified 4 of the 6 *SLX *genes previously identified in the *sgs1*Δ synthetic lethal screen described above [39], as well as three other genes known to show synthetic interactions [39,42-45]. The screen also identified 16 other genes that caused synthetic lethality or sickness when mutated with *sgs1*Δ. One of these genes was *ESC4*. Subsequently, there have been other reports confirming this genetic interaction between *SGS1 *and *ESC4 *[46,47].

Here we demonstrate that the C-terminal two BRCT motifs of Esc4 bind to Sir3 and are sufficient for *SIR*-dependent targeted silencing at *HMR*. Furthermore, the N-terminal four BRCT motifs in Esc4 bind to Slx4, thus linking this DNA repair protein to silent chromatin.

## Results

### Esc4 establishes targeted silencing when targeted to *HMR*

In a screen described previously, we identified Esc4 as a protein that could restore silencing when targeted to an *HMR *locus harboring a deletion of the *HMR-E *silencer [16]. Targeting of proteins to *HMR *was mediated by the binding of a Gal4 DNA binding domain (G_BD_)-hybrid protein to a Gal4 DNA binding site (*G*) that replaced the *HMR-E *silencer (Figure 1). Silencing was assessed using a *URA3 *reporter gene integrated at the *HMR *locus.

### Targeted silencing by Esc4 is *SIR*-dependent

**Figure 2:**
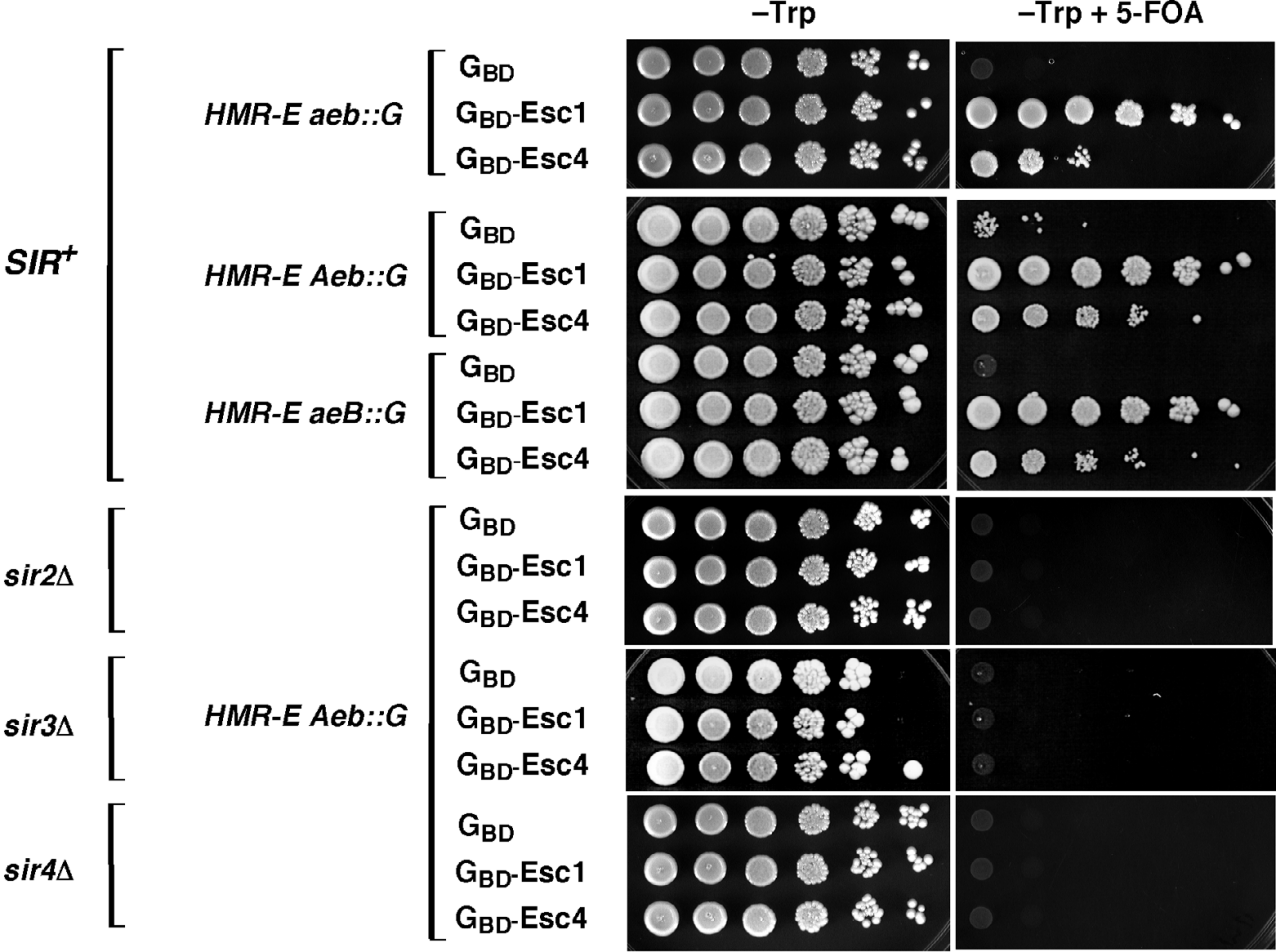
**Targeted silencing by Esc4 is *SIR*-dependent**. Targeted silencing by G_BD_-Esc4 (1–1070) was assessed in *hmr::URA3 *silencing reporter strains containing different *HMR-E *elements replaced by a Gal4 DNA binding site (*G*), similar to the situation shown in Figure 1B. A *TRP1*-marked plasmid expressing G_BD _alone served as a negative control and targeted silencing by the potent targeted silencing factor G_BD_-Esc1(1124–1658) served as a positive control [17]. Targeted silencing by G_BD_-Esc4 in strains with wild-type *SIR *genes (*SIR*^+^) or *sir2*Δ, *sir3*Δ, or *sir4*Δ mutations was examined.

In order to understand how Esc4 promoted silencing when targeted to the *HMR *locus, we tested silencing by G_BD_-Esc4 in various strains. First, targeted silencing by G_BD_-Esc4 was compared to G_BD _alone or to the potent targeted silencing factor G_BD_-Esc1 in the strain in which the screen was performed, which has the entire *HMR-E *silencer deleted and replaced with a Gal4 DNA binding site (*HMR-E aeb::G*) [17]. The fraction of cells silenced by G_BD_-Esc4 was not as great as by G_BD_-Esc1 but, nevertheless, significant targeted silencing at *HMR *was observed despite the complete absence of the *HMR-E *silencer (Figure 2, *HMR-E aeb::G*). When targeted silencing by G_BD_-Esc4 was assessed in strains harboring deletions of just the E and B sites (*HMR-E Aeb::G*) or the A and E sites (*HMR-E aeB::G*) of the *HMR-E *silencer, silencing was increased roughly 50 to 100-fold, as expected for strains with at least one element of the natural silencer (Figure 2).

To test whether targeted silencing by G_BD_-Esc4 was *SIR*-dependent, it was tested in strains deleted for the *SIR2*, *SIR3 *or *SIR4 *genes. As seen in Figure 2, silencing by Esc4 required each of these *SIR *genes. This was expected since targeted silencing by other proteins has been shown to be *SIR*-dependent in every case examined previously [14,16]. It thus seemed likely that Esc4 caused silencing by recruiting the Sir protein complex.

### Saccharomyces Esc4 proteins contain six BRCT motifs and are homologous to *S. pombe *Brc1

**Figure 3:**
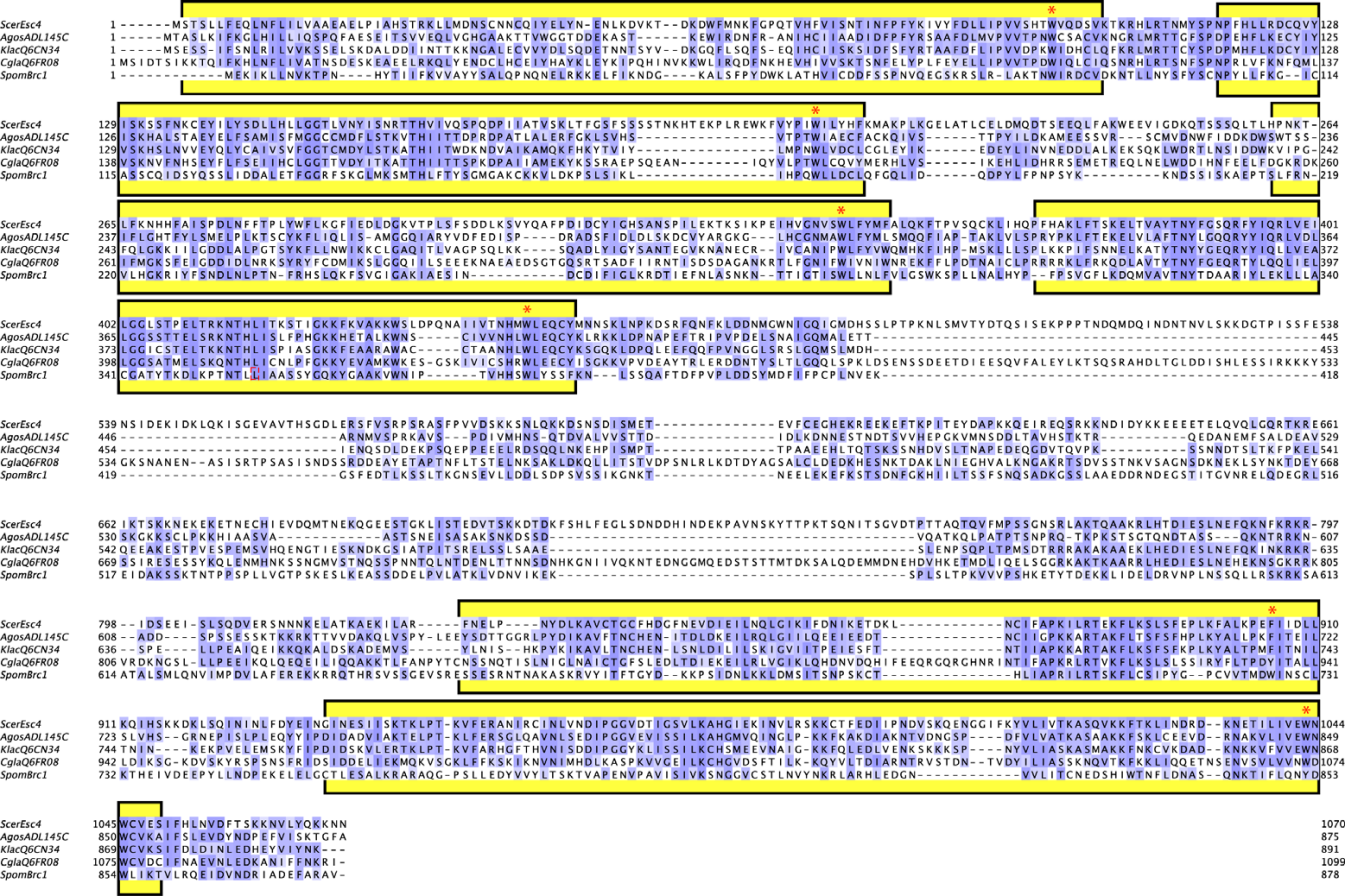
***Saccharomyces cerevisiae *Esc4 has six BRCT motifs that are conserved in both sequence and domain architecture among yeast homologs**. The six BRCT motifs in Esc4 are indicated with brackets and yellow color and were described previously [49]. The motifs in Esc4 are shown as they align with a subset of Esc4 homologs: three from other budding yeasts *Ashbya gossipii*, *Kluyveromyces lactis *and *Candida glabrata *and one from the evolutionarily distant fission yeast *Schizosaccharomyces pombe *Brc1. The highly conserved aromatic residue, a hallmark of BRCT motifs, is marked above the alignment with a red asterisk. Blue color indicates similarity with the predominant residue at the position in the alignment, with darkness correlated with greater similarity as calculated by *ClustalW *software and displayed with *Jalview *alignment editor's BLOSUM62 score viewing option [67, 68].

Esc4 contains several copies of the BRCT motif, originally identified in the human breast cancer susceptibility gene, BRCA1 [23]. The presence of these motifs in Esc4 (encoded by yeast ORF *YHR154w*) has been observed previously by database searches [48,49], although there has been disagreement as to how many BRCTs exist in this protein, with some reports suggesting six BRCTs [49,50] and others only four [21,22,48]. Our own analyses including use of *Pfam *[51,52] confirms the first five putative BRCTs in Esc4, and it seems likely that residues 935–1049, which are BRCT-like with a conserved W or Y residue near the C-terminus, also form a domain that folds into a BRCT type structure as previously reported [49] (Figure 3). Furthermore, analysis of a homolog of Esc4 from *S. pombe*, Brc1, using *Pfam *shows six BRCTs in this homologous protein, with the last two again being the most alternative (E-values of 4.1 × 10^-5 ^and 5.8 × 10^-2 ^for the fifth and sixth, respectively, compared to values ranging from 2.8 × 10^-6 ^to 1.7 × 10^-14 ^for the first four BRCTs), further supporting a conserved total of six motifs in these homologs (Figure 3).

The six BRCT motifs of Esc4 protein exist as a set of four tandem motifs at the N-terminus of the protein and two more at the C-terminus (Figure 3, yellow lines). These two sets of BRCT motifs are separated by a 375 amino acid linker region. Although there are proteins from various budding yeasts with compelling sequence similarity to *S. cerevisiae *Esc4 (e.g. *Ashbya gossipii*, *Kluyveromyces lactis*, and *Candida glabrata*, as shown in Figure 3), currently the only obvious non-budding yeast homolog candidate is Brc1 from the evolutionarily distant fission yeast *Schizosaccharomyces pombe *(Figure 3, bottom line). *S. pombe *Brc1 is 878 amino acids in length and also contains four BRCTs at the N-terminus and two at the C-terminus, separated by a linker region. Esc4 and Brc1 are 52% similar and 21% identical overall. Human and mouse PTIP proteins have been reported to be quite similar in sequence [21,53], but do not contain the classically-conserved C-terminal aromatic residue present in all of the six BRCTs highlighted with a red asterisk in the alignment shown in Figure 3. Therefore, it is not yet clear if these proteins are *bona fide *Esc4/Brc1 homologs.

### The C-terminal two BRCT motifs of Esc4 are sufficient for targeted silencing

**Figure 4:**
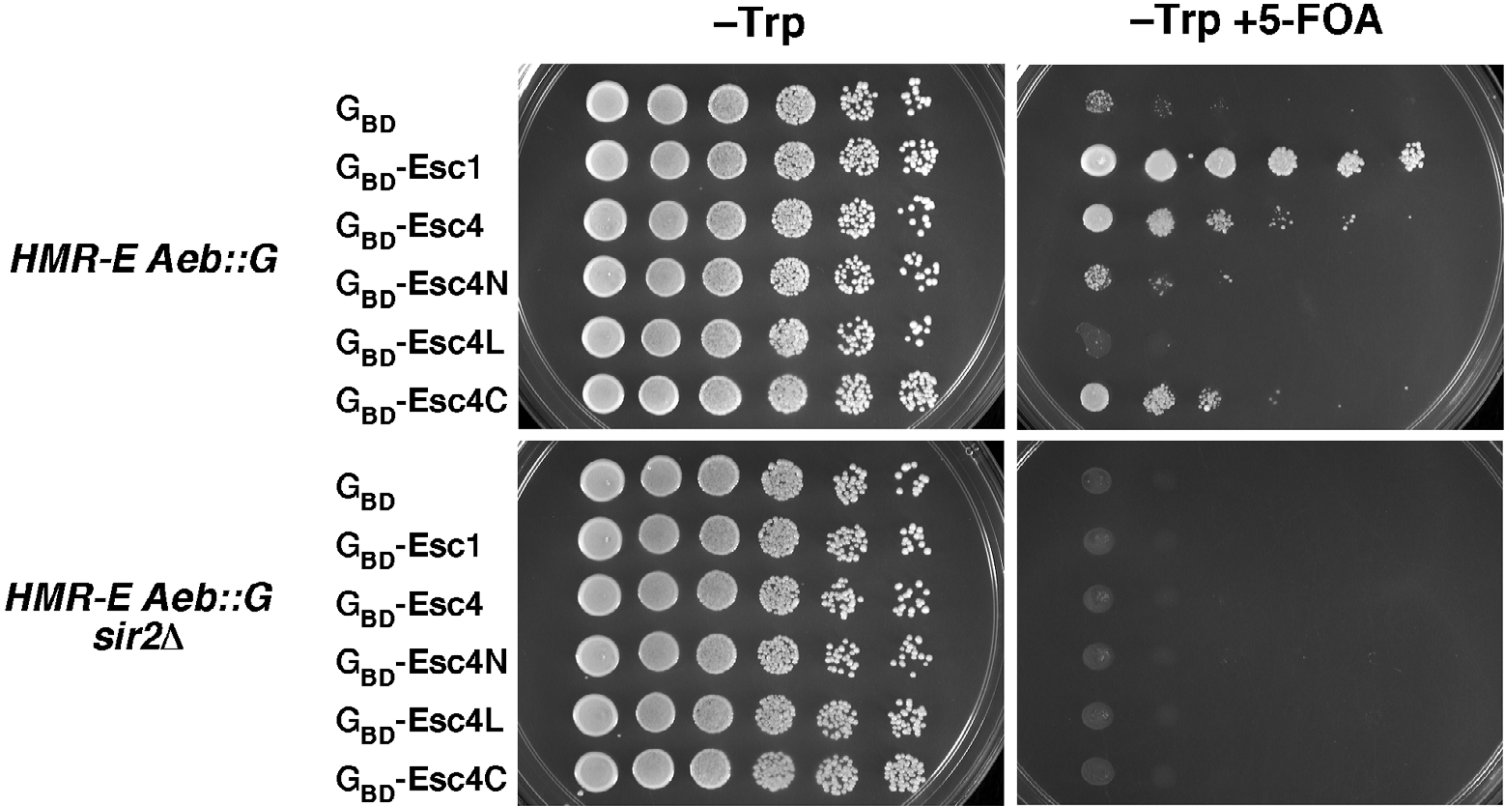
**Targeted silencing activity is achieved by the C-terminus of Esc4**. Three different regions of Esc4, containing either the first four BRCT motifs (1–480), linker region (480–836), or last two BRCTs (818–1070) were tested for targeted silencing in a strain with an *HMR-E Aeb::G *silencer and *URA3 *reporter gene at *HMR*. GBD-Esc1 served as a potent positive control and GBD as a negative control. *SIR2*-dependence of targeted silencing was also determined.

The Esc4 hybrid protein isolated in our targeted silencing screen was full-length [16] and therefore contained all six predicted BRCT motifs. To determine which part of Esc4 and which BRCT motifs were responsible for the *SIR*-dependent targeted silencing, we constructed three G_BD _hybrids: one to the N-terminal four BRCT motifs (G_BD_-Esc4N, aa 1–480), one to the linker between the N- and C-terminal sets of motifs (G_BD_-Esc4L, aa 480–836), and one to the C-terminal two BRCT motifs (G_BD_-Esc4C, aa 818–1070). These constructs were tested for targeted silencing in a strain harboring deletions of the E and B sites (*HMR-E Aeb::G*) and in a *sir2*Δ derivative of that strain (Figure 4; see Table 1 for strain information). Significant targeted silencing was observed by G_BD_-Esc4C, although it was not as much as with full-length Esc4. The observed silencing by G_BD_-Esc4C was *SIR*-dependent, as observed for the full-length protein (Figure 2). No significant silencing was seen with Esc4N or with the linker region, Esc4L (Figure 4).

### The C-terminal BRCT motifs of Esc4 interact with Sir3

Because the C-terminal two BRCT motifs of Esc4 gave targeted silencing, we suspected that this region of the protein was binding to a silencing protein to recruit the Sir complex. Using a LexA-Esc4C hybrid and the two-hybrid reporter strain L40 [54], we tested for two-hybrid interactions with several Gal4 activation domain (GAD)-silencing protein constructs, including Sir1, Sir2, Sir3, Sir4 and Rap1 [3,17,55]. A strong interaction (i.e., the *lacZ *reporter gene generated blue color from X-gal visible in 15 minutes) with GAD-Sir3 (aa 252–978) was identified, as well as a weaker interaction with GAD-Sir4 (aa 839–1358) (Table 2). None was detected with Sir1, Sir2 or Rap1. Because the GAD-Sir4 (aa 839–1358) hybrid contained the region known to bind to Sir3 [7], we hypothesized that LexA-Esc4 was binding to GAD-Sir4 via a bridge of endogenous Sir3. To test this, we used a derivative of strain L40 harboring a *sir3*Δ mutation and examined the LexA-Esc4 interaction with GAD-Sir4. In this case, the interaction with GAD-Sir4 was no longer observed, whereas the interaction with GAD-Sir3 and an unrelated two-hybrid control interaction were unaffected (Table 2). When a *sir4*Δ derivative of L40 was used, no change in the LexA-Esc4 interactions with GAD-Sir3 or GAD-Sir4 was observed, further supporting the idea that Esc4 requires Sir3 to interact with Sir4, and not vice versa. Taken together, the targeted silencing data strongly suggest an interaction (probably a direct one) between the C-terminal BRCT motifs and the silencing protein Sir3.

### The N-terminal four BRCT motifs of Esc4 bind to Slx4

The above results indicated that Esc4 caused *SIR*-dependent targeted silencing primarily by binding to Sir3 through its C-terminal two BRCT motifs. In addition, Esc4 also contains four BRCT motifs at its N-terminus and these BRCT motifs are more similar to those found in various proteins from diverse eukaryotes (Figure 3, and ref. [49]). In order to identify proteins that bind to these N-terminal BRCT motifs, a two-hybrid screen was performed using a LexA-Esc4N (aa 1–480) fusion protein. Strikingly, from screening ~2 × 10^7 ^library plasmids expressing GAD protein hybrids, thirteen clones containing in-frame fusions of GAD to Slx4 were identified (Table 3). In-frame GAD fusions to the six different positions in Slx4 were isolated (see Table 3) and all contained at least the C-terminal half of the protein (residues 383–748). The Esc4N-Slx4C two-hybrid interaction was a very strong one and was specific. Also, Slx4 did not show an interaction with Esc4C. Thus, the N-terminal BRCT motifs of Esc4 are not only necessary for binding Slx4, as reported while this manuscript was in preparation [22], but are indeed sufficient for this interaction *in vivo*. Furthermore, our two-hybrid data show that the region of Slx4 sufficient for Esc4 binding resides in its C-terminus.

### Genetic and phenotypic analysis of *ESC4*

A heterozygous diploid strain with a complete deletion of *ESC4 *was constructed and dissected to generate a null mutant haploid. This *esc4*Δ mutant grew normally and also mated with normal efficiency (suggesting no gross defect in *HM *silencing). Furthermore, when an *esc4*Δ mutation was introduced into a strain with a telomere reporter gene, no telomeric silencing defect was seen (data not shown). Thus, although Esc4 binds to Sir3, Esc4 does not appear to be a protein required for Sir protein-mediated silencing.

**Figure 5:**
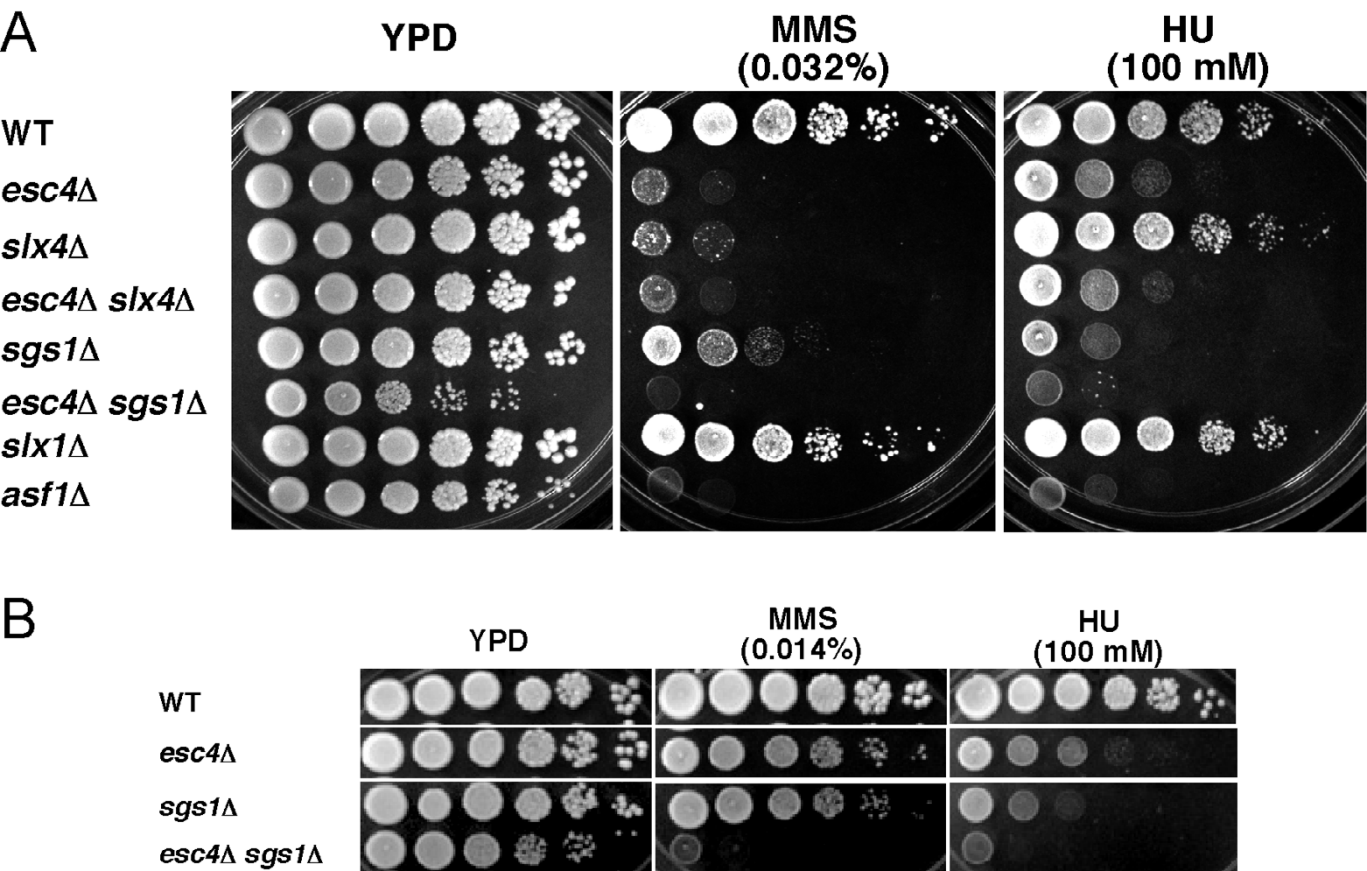
**Genetic evidence that *ESC4 *and *SLX4 *function together in a parallel pathway to *SGS1 *for repair of damaged DNA**. Strains were grown in YPD medium to saturation and then ten-fold serial dilutions were spotted onto YPD, YPD + HU (100 mM), or YPD + MMS (0.032% in part A and 0.014% in part B) media. Cells were incubated at 30°C for 2 days (SC and SC + MMS) or 3 days (SC + HU) before being photographed. WT, wild type. An *asf1*Δ mutant was used as a positive control for MMS and HU sensitivity.

The BRCT motif was originally identified in the human BRCA1 tumor suppressor protein [23]. BRCA1 functions in DNA repair and DNA damage-sensing in cell cycle checkpoints (reviewed in [56]). As shown in Figure 5, strains deleted for *ESC4 *grew significantly less well than wild type on medium containing either MMS or HU. This result confirms reports that have since been published [18,21,22,46], as does our observation that *esc4*Δ mutants are not sensitive to ultraviolet radiation [18,21] (data not shown).

As shown in Figure 5, like *esc4*Δ mutants, *slx4*Δ mutants were sensitive to 0.032% MMS. Furthermore, the *esc4*Δ *slx4*Δ double mutant did not exhibit a greater MMS sensitivity than either single mutant (Figure 5A and data not shown), suggesting that they cooperate in providing resistance to MMS. Another group reported that an *esc4*Δ *slx4*Δ double mutant was more sensitive than either single mutant but the difference was very slight [22]. In contrast, an *esc4*Δ, but not a *slx4*Δ mutant, was significantly HU-sensitive, and the double mutant was no more sensitive than the *esc4*Δ strain. Thus, Esc4 appears to act independently of Slx4 in providing resistance to HU.

Because Esc4 bound to Slx4 and because the mutant was sensitive to MMS, this suggested that Esc4 might function in the same pathway as Slx4. *SLX4 *was first identified in a screen for genes required for viability of yeast cells deleted for *SGS1 *[39]. Therefore, we tested if *esc4 *was also synthetically lethal with *sgs1*. To do this, an *esc4*Δ mutant was crossed with an *sgs1*Δ mutant, the diploid was sporulated and dissected and meiotic progeny were analyzed. Haploid *esc4*Δ *sgs1*Δ cells were viable, but were noticeably slower-growing that either single mutant (e.g., see Figure 5, YPD plate). While this work was in progress, this genetic interaction was also observed in genome-wide studies [41,46].

An *sgs1*Δ mutant showed sensitivity to both MMS and HU (Figure 5), as expected based on previously published results. An *asf1*Δ mutant was used as a control and displayed sensitivity to both DNA damaging chemicals, as expected [57,58]. Interestingly, an *esc4*Δ *sgs1*Δ mutant displayed MMS and HU sensitivity that was much more pronounced than that of either single mutant (Figure 5A and 5B). The enhanced sensitivity of this double mutant (10,000-fold on 0.014% MMS plates) seemed to be due to a synergistic repair defect and not entirely due to the growth defect (10-fold difference in colony number on YPD control plates) that was also observed in the *esc4*Δ *sgs1*Δ strain.

## Discussion

By screening a library of factors that could function in place of the *HMR-E *silencer when targeted to DNA, we identified Esc4 for its ability to establish silent chromatin [16]. Protein sequence analysis showed that Esc4 protein contains six BRCT motifs; four are found in tandem at the amino-terminus and two more are at the carboxy-terminus. The entire Esc4 protein was present in the hybrid identified in the targeted silencing screen. Since targeted silencing by Esc4 at *HMR *was found to be *SIR*-dependent, it seemed likely that some region within Esc4 was attracting a silencing protein complex to DNA. We tested subsets of the BRCT motifs, as well as the linker between them, for targeted silencing at *HMR*. These experiments demonstrated that the C-terminal two BRCTs caused targeted silencing that was nearly as strong as with full-length Esc4. Because silencing by this pair of BRCT motifs of Esc4 was also *SIR*-dependent, it seemed very likely that this region was recruiting a Sir protein when tethered to DNA. Therefore, we tested the C-terminal BRCT motifs for interactions with known silencing proteins by two-hybrid analysis. We identified a specific interaction with Sir3 (aa 252–978). We conclude that binding of Sir3 by Esc4 is likely to be responsible for the *SIR*-dependent targeted silencing activity.

In some cases BRCT motifs have been shown to bind to phosphorylated serine residues. Specifically, they have been shown to bind to phosphopeptides with the following consensus: pSxxF [32]. Interestingly, Sir3 has an SxxF sequence (aa 583–586) within the Esc4-interacting region that we describe here (aa 252–978) and, furthermore, Sir3 protein has been shown to be phosphorylated [59,60], suggesting that an Esc4 BRCT motif or perhaps the combination of the two in the C-terminus may bind to phospho-Sir3. However, not all proteins bound by BRCT motifs have the SxxF motif [61], so the precise BRCT-interacting region of Sir3 could be elsewhere.

In addition to binding Sir3 via C-terminal tandem BRCT motifs, Esc4 also binds to Slx4 via four tandem N-terminal BRCTs, as we have shown here by two-hybrid screening. This two-hybrid result demonstrates that these four BRCTs are sufficient for binding Slx4 and agrees with a recent report showing that the N-terminal BRCT motifs are required for this interaction [22]. It seems quite possible that Esc4 could bind Sir3 and Slx4 concurrently, given that these nuclear proteins' binding sites within Esc4 map to BRCT clusters separated by a long linker. Slx4 has been shown to heterodimerize with the endonuclease Slx1 to cleave DNA containing 5' -flap structures, such as in stalled replication forks, to facilitate their repair [62]. Thus, Esc4 binds the silencing protein Sir3 and also to Slx4, an important DNA repair complex component. Esc4 may play a role in facilitating repair of aberrant DNA structures, perhaps specifically within silent chromatin. Esc4 is a Mec1 kinase target [21] and this phosphorylation is required for its repair function. It is possible that phosphorylation of Esc4 by Mec1, which occurs just N-terminal to the Sir3-binding BRCTs, regulates association with Sir3 or other factors required for its ability to repair particular chromosomal loci in S phase.

We analyzed Esc4 protein alignments (such as that shown in Figure 3) for evidence of conserved regions in the protein other than BRCT motifs. One region of interest was the SQ/TQ motifs between amino acids 743 and 807, which were shown to be important for function in DNA repair [21]. We did not find that these motifs were well-conserved, suggesting that the specific site of phosphorylation is not particularly critical in proteins with otherwise similar overall BRCT domain architecture (i.e., [BRCT]_4_-linker-[BRCT]_2 _arrangement). This may be because of some differences in Esc4 functions in diverse yeasts or may suggest that flexibility is tolerated in positioning of the phosphorylation sites, and therefore the exact relative location of kinase target sites has not been constrained during evolution.

Future structural and genome sequencing studies are likely to unveil similarities and differences between multi-BRCT domain containing proteins. Whether these proteins play largely protein-scaffolding roles or also contain intrinsic enzymatic properties will be interesting to discover.

## Conclusion

We have shown that Esc4 caused targeted silencing when tethered at a weakened *HMR *locus. The targeted silencing activity was primarily due to the C-terminal two tandem BRCT motifs in Esc4, which bound to Sir3, probably through a direct interaction. This interaction led to the recruitment of the Sir complex and hence caused targeted silencing. The N-terminal BRCT domains were sufficient for binding to Slx4, which functions with Esc4 in DNA repair. Thus, the nuclear Esc4 protein uses its six BRCT motifs to connect diverse proteins involved in DNA repair and silent chromatin.

## Methods

### Targeted silencing

Esc4 was identified in a targeted silencing screen that has been described previously [16]. Briefly, a Gal4 DNA binding domain (G_BD_) library was screened for hybrid proteins capable of establishing silencing of a *URA3 *reporter gene integrated in place of mating-type genes at an *HMR *locus that had the *HMR-E *silencer replaced by a Gal4 DNA binding site (*G*) (see Figure 1A). A full length G_BD_-Esc4 (1–1070) clone, aeb15, was identified as being capable of establishing targeted silencing of *hmr::URA3*, causing resistance to 5-fluororotic acid (5-FOA). This G_BD_-Esc4 clone was subsequently transformed into strain YEA76 (*HMR-E Aeb::G hmr::URA3 gal4::LEU2*) and YEA77 (*HMR-E aeB::G hmr::URA3 gal4::LEU2*) (see Table 1 and Yeast Strain section for details) and tested for targeted silencing. To test *SIR*-dependence of targeted silencing by Esc4 at *HMR*, shown in Figure 4, targeted silencing in strain YEA76 (*SIR*^+^) was compared with that in *sir *mutant derivatives YAM7 (*sir2*Δ), YEA 118 (*sir3*Δ), and YKAB17 (*sir4*Δ).

For the targeted silencing experiments shown in Figures 2 and 4, assays were carried out as follows: strains were transformed with plasmids expressing the appropriate G_BD _hybrid protein, grown at 30°C for two days in SC -Trp medium (to select for the G_BD _plasmid), serially diluted ten-fold five times and spotted on SC -Trp + 5-FOA plates (to assay for silencing at *HMR*) or on SC -Trp control plates.

### Plasmids

Plasmid aeb15, expressing G_BD_-Esc4(1–1070) was isolated in the targeted silencing screen. This plasmid was recovered from a library based on pGBT9.C (*ADH1*_*UAS*_-G_*BD*_*, TRP1, CEN4/ARS1*; see Acknowledgements). To generate plasmids for use in the two-hybrid system, *ESC4 *sequences were amplified from genomic DNA and subcloned into plasmid pSTT91, a derivative of pBTM116 that contains the *ADE2 *gene ([63]; see also [64]). Plasmids pAM2 (LexA-Esc4N (a.a. 1–480) and pAM7 (LexA-Esc4C (a.a. 818–1070) were used for two-hybrid experiments. To test for LexA-Esc4C binding to GAD-Sir3 and GAD-Sir4, plasmid pEDA195, GAD-Sir3(252–978), and pCTC36, GAD-Sir4(839–1358), were used. pCTC36 expresses the same region of *SIR4 *as plasmid pCTC18 [55] except that the Sir4 hybrid is expressed from pGAD-R. pEDA195 was constructed by cloning a *Pst*I fragment of the *SIR3 *gene into vector pGAD424. Plasmid pAM2, expressing LexA-Esc4(1–480), and a clone expressing GAD-Slx4(383–748) isolated in the two-hybrid screen were used as a positive control in two-hybrid experiments summarized in Table 3.

### Yeast strain construction

All strains are listed in Table 1. To make *slx1*Δ, *slx4*Δ, *sir2*Δ, *sir4*Δ, and *sgs1*Δ mutants, PCR primers with 5' homology to sequences flanking these ORFs and 3' homology to sequences in a plasmids harboring selectable marker genes were used for PCR, generating targeting cassettes that were transformed into yeast as has been previously described [65]. *esc4*Δ *::his5*^+ ^mutants were generated by the same method, but using a different plasmid as a template for PCR (gift of N. Dean, Stony Brook University). Strain YEA76 and its derivatives are derived from strain YSB35 [13].

### Two-hybrid screening and direct tests

Screening was performed essentially as described [54,63]. Plasmid pAM2, which expresses LexA-Esc4(1–480), (*ADE2, TRP1, 2*μ) was co-transformed with approximately 1 μg of GAD library (*LEU2, 2*μ; [66]) into strain L40 [54], which contains LexA binding sites upstream of both the *HIS3 *gene and the *LacZ *gene. The following specificity tests were performed: (1) His^+ ^candidates were assayed for *lacZ *expression both after curing candidates of either the LexA or the GAD plasmid, (2) GAD hybrids were tested for interactions with nonspecific LexA hybrids (e.g. LexA-lamin) by mating candidates cured of the bait plasmid to LexA-containing AMR70 [54], and (3) the GAD hybrid plasmids which passed the aforementioned tests were subsequently retransformed into L40 along the original bait and tested for LacZ expression.

Two-hybrid tests done using the C-terminal BRCT motifs in Esc4 were performed by co-transforming L40 with plasmid pAM7, expressing LexA-Esc4(818–1070), and various GAD hybrids to silencing proteins, such as Sir1, Sir2, Sir3, Sir4, and Rap1. To test whether Sir3 bridged binding of LexA-Esc4(818–1070) to Sir4, a *sir3Δ::kanMX6 *derivative of L40 was generated (see yeast strains section for details), strain YRJ3, and the interaction was then retested in this strain. The control *sir4*Δ-derivative of L40 used was strain YJL03.

### DNA damage sensitivity tests

Cultures were grown in YPD medium ~18 h at 30°C and then serially diluted ten-fold, five times before being spotted onto plates containing YPD medium with MMS, HU, or no chemical. Cells were then incubated at 30°C and, in the case of MMS media, the plates were wrapped in aluminum foil.

## Authors' contributions

RS and DCZ initiated and oversaw the course of the study, designed the experiments and wrote the paper. DCZ did the targeted silencing tests, protein sequence alignments and made the figures. ASRM and DCZ performed the two-hybrid screening and direct two-hybrid tests. JJC and RAJ did some of the genetic analyses. All authors have read and approved this manuscript.

## Figures and Tables

**Table 1:** Yeast strains used in this study

Strain	Genotype	Reference or Source
W303-1a	*MAT****a****leu2–3,112 ura3-1 his3–11,15 trp1–1 ade2-1 can1–100*	R. Rothstein
W303-1b	*MAT*α version of W303-1a	R. Rothstein
W303	Diploid from W303-1a × W303-1b	This study
YDZ5	W303 *MAT****a****/MAT*α *esc4Δ::his5*^+^*/ESC4*	This study
YDZ174	W303 *MAT****a****/MAT*α *esc4Δ::his5*^+^*/esc4Δ::his5*^+^	This study
YDZ3	W303-1a *esc4*Δ*::his5*^+^	This study
YDZ4	W303-1b *esc4*Δ*::his5*^+^	This study
YEA78	W303-1b *hmr::URA3 HMR-E (aeb::G) gal4::LEU2*	This study
YEA76	YEA78 but *HMR-E (Aeb::G)*	This study
YEA77	YEA78 but *HMR-E (aeB::G)*	Thi study
YAM7	YEA76 *sir2*Δ*::kanMX6*	This study
YEA118	YEA76 but *GAL4 sir3::LEU2*	This study
YKAB17	YEA76 *sir4*Δ*::kanMX6*	This laboratory
YDS631	W303-1b *adh4::URA3*-(C_1–3_A)_*n*_	[13]
YDZ22	YDS631 *esc4*Δ*::his5*^+^	This study
YAM1	W303-1a *slx4*Δ*::his5*^+^	This study
YAM2	W303-1b *slx4*Δ*::his5*^+^	This study
YAM3	W303-1a *slx4*Δ*::kanMX6*	This study
YAM4	W303-1b *slx4*Δ*::kanMX6*	This study
YAM13	W303-1b *sgs1*Δ*::kanMX6*	This study
YDZ243	W303-1_ *sgs1*Δ*::kanMX6 esc4*Δ*::his5+*	This study
YAM6	W303-1a *esc4*Δ*::his5*^+^*slx4*Δ*::kanMX6*	This study
YAS501	W303-1a *asf1*Δ*::his5*^+^	This laboratory
L40	*MAT****a****his3*Δ*200 trp1–901 leu2–3,112 ade2 URA3::(lexAop)*_8_*-lacZ LYS2::(lexAop)*_4_*-HIS3*	[54]
YRJ3	L40 *sir3*Δ*::kanMX6*	This study
YJL103	L40 *sir4*Δ*::kanMX6*	This study

**Table 2:** Esc4 binds to Sir3 in the two-hybrid system

		Interaction in two-hybrid strains^a^		
LexA hybrid	GAD hybrid	L40	L40 Δ *sir3*	L40 Δ *sir4*

LexA-Esc4 (818–1070)	GAD-Sir3 (252–978)	++	++	++
LexA-Esc4 (818–1070)	GAD-Sir4 (839–1358)	+	-	+
LexA-Esc4 (818–1070)	GAD vector	-	-	-
LexA-Esc4 (1–480)	GAD-Slx4 (383–748)	+++	+++	+++

^a ^Three +++ signs means an easily visible blue color from the *lacZ *reporter gene was visible within 15 minutes, whereas two ++ signs means it was visible within 30 minutes and one + sign means it was visible between 40 minutes and two hours.

**Table 3:** The amino-terminal BRCT motifs of Esc4 bind to Slx4

LexA hybrid	GAD hybrid	Number of isolates
LexA-Esc4 (1–480)	GAD-Slx4 (166–748)	1
LexA-Esc4 (1–480)	GAD-Slx4 (194–748)	2
LexA-Esc4 (1–480)	GAD-Slx4 (260–748)	1
LexA-Esc4 (1–480)	GAD-Slx4 (280–748)	4
LexA-Esc4 (1–480)	GAD-Slx4 (368–748)	3
LexA-Esc4 (1–480)	GAD-Slx4 (383–748)	2
